# Sinus Surgery Improves Maxillary Sinus Deformation in Cystic Fibrosis Detected by Magnetic Resonance Imaging

**DOI:** 10.1177/19458924261435339

**Published:** 2026-04-06

**Authors:** Miray-Su Yılmaz Topçuoğlu, Ingo Baumann, Veronika Kolb, Hans-Ulrich Kauczor, Olaf Sommerburg, Mirjam Stahl, Marcus A Mall, Monika Eichinger, Lena Wucherpfennig, Mark O Wielpütz

**Affiliations:** 1 Department of Otorhinolaryngology, Head and Neck Surgery, Medical Faculty Heidelberg, 27178 University Hospital Heidelberg, Heidelberg, Germany; 2 Department of Diagnostic and Interventional Radiology, 27178 University Hospital Heidelberg, Heidelberg, Germany; 3 Translational Lung Research Center Heidelberg (TLRC), 542891 German Center for Lung Research (DZL), Heidelberg, Germany; 4 Department of Diagnostic and Interventional Radiology With Nuclear Medicine, Thoraxklinik at University Hospital Heidelberg, Heidelberg, Germany; 5 Department of Translational Pulmonology and Division of Pediatric Pulmonology and Allergy and Cystic Fibrosis Center, University of Heidelberg, Heidelberg, Germany; 6 Department of Pediatric Respiratory Medicine, Immunology and Critical Care Medicine, Charité – Universitätsmedizin Berlin, Corporate Member of Freie Universität Berlin and Humboldt-Universität zu Berlin, Berlin, Germany; 7 German Center for Lung Research (DZL), associated partner site Berlin, Berlin, Germany; 8 Berlin Institute of Health at Charité – Universitätsmedizin Berlin, Berlin, Germany; 9 German Center for Child and Adolescent Health (DZKJ), Partner Site Berlin, Berlin, Germany; 10 Department of Radiology and Neuroradiology, Greifswald University Hospital, Greifswald, Germany

**Keywords:** cystic fibrosis, chronic rhinosinusitis, magnetic resonance imaging, maxillary sinus, mucopyoceles, outcome, prevalence, sinus dimensions, sinus surgery, scores

## Abstract

**Background:**

Chronic rhinosinusitis (CRS) contributes to morbidity in cystic fibrosis (CF), and sinus surgery serves as second-line treatment. Magnetic resonance imaging (MRI) was recently shown to differentiate CF-related CRS (CF-CRS) manifestations, and to monitor therapy response, but has not been used to investigate the effects of sinus surgery on CF-CRS.

**Objective:**

The aim of this study was to systematically study the effects of sinus surgery on CF-CRS.

**Methods:**

Twenty controls with CF (median age 15 years, range 9-33 years) who had not undergone sinus surgery with annual MRI examinations were age-matched to the surgery group. The surgery group comprised 10 individuals with CF (median age 15 years, range 8-32 years) who underwent endoscopic sinus surgery between 2010 and 2018 and underwent MRI in median 2.5 months before (MRI1) and at least one (MRI2) or two (MRI3) annual MRIs after surgery. The median time difference between sinus surgery and MRI2 was 14 months, and between MRI2 and MRI3 it was 13 months. All patients were modulator-naïve. The established CRS-MRI score was used including sinus dimension measurement.

**Results:**

In controls, the median maxillary sinus width was stable from MRI1 through MRI3 (range 23.0-24.5 mm; *P* > .999). In the surgery group, the median maxillary sinus width decreased from MRI1 to MRI2 (−5.5 mm, *P* < .01), and remained stable from MRI2 to MRI3 (+3.0 mm; *P* = .544). The prevalence of maxillary sinus deformation decreased from MRI1 to MRI2 (−35%; *P* < .05) and was stable from MRI2 to MRI3 (+19%; *P* = .295). The CRS-MRI sum score was stable from MRI1 through MRI3 in controls (median 28, 23 and 34 at MRI1-2-3), and in the surgery group (36, 35 and 39, respectively) (*P* = .743-.999).

**Conclusion:**

Sinus surgery improves maxillary sinus width and deformation. The CRS-MRI score could not detect further benefits of surgery on CF-CRS. MRI supports the evaluation of sinus surgery in the era of modulator treatment strategies as some patients still suffer from CF-CRS despite optimized modulator treatment and there is a need to identify patients that still might profit from sinus surgery.

## Introduction

Chronic rhinosinusitis (CRS), a highly prevalent condition in individuals with cystic fibrosis (CF), causes chronic functional impairment and reduced quality of life.^1–4^ As life expectancy for individuals with CF increases, the focus is expanding to comorbidities such as CF-related CRS (CF-CRS).^5,6^ In particular, the maxillary sinus exhibits signs of CRS from infancy on cross-sectional imaging.^7–9^ Magnetic resonance imaging (MRI) provides a detailed monitoring of CF-CRS without radiation exposure^9–13^ and a CRS-MRI scoring system has recently been established.^10^ This allows for the semiquantitative longitudinal monitoring of the sinuses and the differentiation of sinus abnormalities in patients with CF-CRS.^9^ Prior to the era of cystic fibrosis transmembrane conductance regulator (CFTR) modulators (CFTRm), the most commonly utilized therapeutic modalities for CF-CRS were either symptomatic approaches such as nasal irrigation or nebulization with isotonic or hypertonic sodium chloride solution, or, in cases of severe symptoms, endoscopic sinus surgery as second line treatment modality.^1,14–17^ Individuals suffering from CF-CRS often require repeated intervention, even subsequent to extensive sinus surgery.^17–19^ The actual effects of surgery on the postoperative course of CF-CRS have not been systematically studied with imaging. Therefore, the aim of the present study was to investigate the effects of endoscopic sinus surgery on CF-CRS in patients employing annual MRI in combination with the established CRS-MRI scoring system.^10^

## Materials and Methods

### Study Population

**Figure 1.**
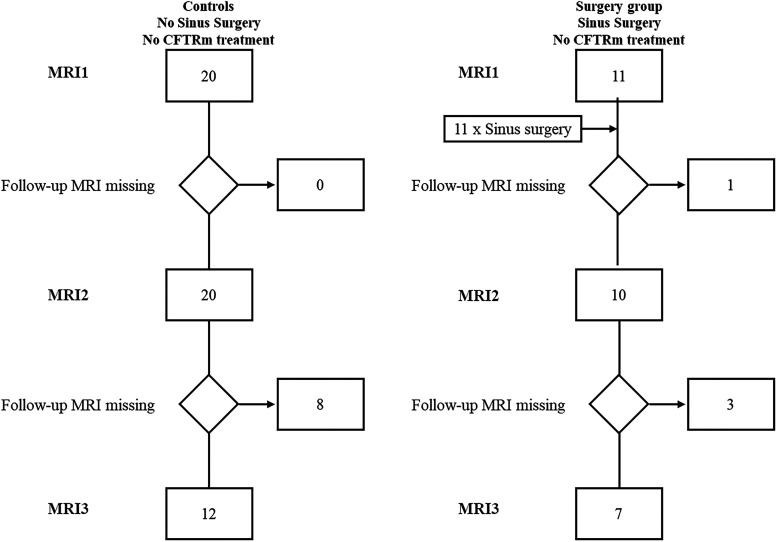
Study flowchart.

Abbreviations: CFTRm, cystic fibrosis transmembrane conductance regulator modulator; MRI, magnetic resonance imaging.

This study is part of an ongoing prospective observational study (clinicaltrials.gov identifiers NCT00760071, NCT02270476 and DRKS00031784) and has been approved by the institutional ethics committee of the Medical Faculty of the University Heidelberg (S-211/2011, S-370/2011, S-646/2016). All patients/their legal guardians provided written informed consent. All enrolled patients were CFTRm naïve. The diagnosis of CF was based on increased sweat chloride concentrations (≥60 mmol/l), *CFTR* mutation analysis, and in pancreatic sufficient patients with borderline sweat test results (Cl^−^ 30-60 mmol/l) by assessment of CFTR protein function in rectal biopsies^20,21^ (Supplemental Table E1). Twenty controls with CF (median age 15 years, range 9-33 years) who had not undergone sinus surgery with two (*n* = 20) or three (*n* = 12) annual MRI examinations (MRI1, MRI2, MRI3) were age-matched (age at MRI1) to the surgery group in a 1:2 ratio (Table 1, Figure 1). Of the 20 controls, 13 patients had a nasal swab at the time of MRI1, and all of these were negative for Pseudomonas aeruginosa. However, three of these patients had positive Pseudomonas results in their sputum and/or throat swabs. Of the other seven controls without nasal swabs, five had sputum samples (three of these were positive for Pseudomonas) and two had throat swabs (both were negative for Pseudomonas) at the time of MRI1.

**Table 1.** Patient Baseline Characteristics.

	**Controls**	**Surgery Group**
***N***	20	10
**Age (yr)**	15; 9-33; 21	15; 8-32; 19
**Gender (m/f)**	9/11	5/5
***CFTR* genotype, *n* (%)**		
**F508del/F508del**	8 (40)	2 (20)
**F508del/other**	11 (55)	6 (60)
**Other/other**	1 (5)	2 (20)
**Pancreatic insufficiency, *n* (%)**	18 (90)	8 (80)
**Intake of CFTRm, *n* (%)**	0 (0)	0 (0)
**Pseudomonas detection, *n* (%)**		
**Positive intraop. sinus samples**	–	1 (10)
**Positive nasal swabs at MRI1**	0 (0)	–
**Positive sputum/throat swabs at MRI1**	6 (30)	2 (20)

Data were collected at the time of the first magnetic resonance image MRI1. Abbreviations: yr, years; m, male; f, female; CFTR, cystic fibrosis transmembrane conductance regulator; CFTRm, CFTR modulator. Pseudomonas detection refers to identified Pseudomonas aeruginosa from nasal or sputum and/or throat swabs at MRI1 for the controls, and for the study group from intraoperative (intraop.) sinus samples, sputum and/or throat swabs. In controls, 13 patients had documented nasal samples, and seven patients only had documented sputum and/or throat samples. In the surgery group, all ten sinus samples were taken intraoperatively. No nasal swabs at MRI1 were taken in these patients. Sputum and/or throat samples at MRI1 were documented in all ten patients of the study group. Age presented as median; minimum-maximum; interquartile range. Gender distribution is given in absolute numbers. All other data are given as absolute numbers *n* with percentage given in brackets.

The surgery group comprised ten individuals with CF (median age 15 years, range 8 to 32 years) who underwent endoscopic sinus surgery, performed by one senior surgeon I.B., at the Department of Otorhinolaryngology between 2010 and 2018 (Table 1, Figure 1). They underwent one MRI in median 2.5 months (range: 0-62; interquartile range (IQR): 5.5) before (MRI1) and at least one (MRI2, *n* = 10) or two annual MRIs (MRI3, *n* = 7) after surgery. The median time difference between sinus surgery and MRI2 was 14 months (range: 4-54; IQR: 22), and between MRI2 and MRI3 it was 13 months (range: 2-19; IQR: 12). The decision to perform sinus surgery was primarily based on the patient's clinical symptoms, and the expected outcome of the surgery was discussed thoroughly with the patient prior to surgery. Although imaging was supportive, it was not the decisive factor. Endoscopic sinus surgery as semi-radical approach was performed, with the creation of broad sinus openings.^17,22^ No turbinate resections, medial maxillectomies or Draf procedures were performed. The surgery group received sputum and/or throat swabs at MRI1, as well as intraoperative sinus samples; no additional nasal swabs were taken at MRI1. One patient had a positive sputum sample prior to surgery at MRI1, as well as a positive Pseudomonas aeruginosa sinus sample taken intraoperatively. Of the other nine patients, one was positive in the sputum sample at MRI1, but not in the intraoperative sinus sample. All the other patients in the study group tested negative for Pseudomonas aeruginosa in sputum and/or throat samples taken at MRI1, and in intraoperative sinus samples.

### Magnetic Resonance Imaging

**Figure 2.**
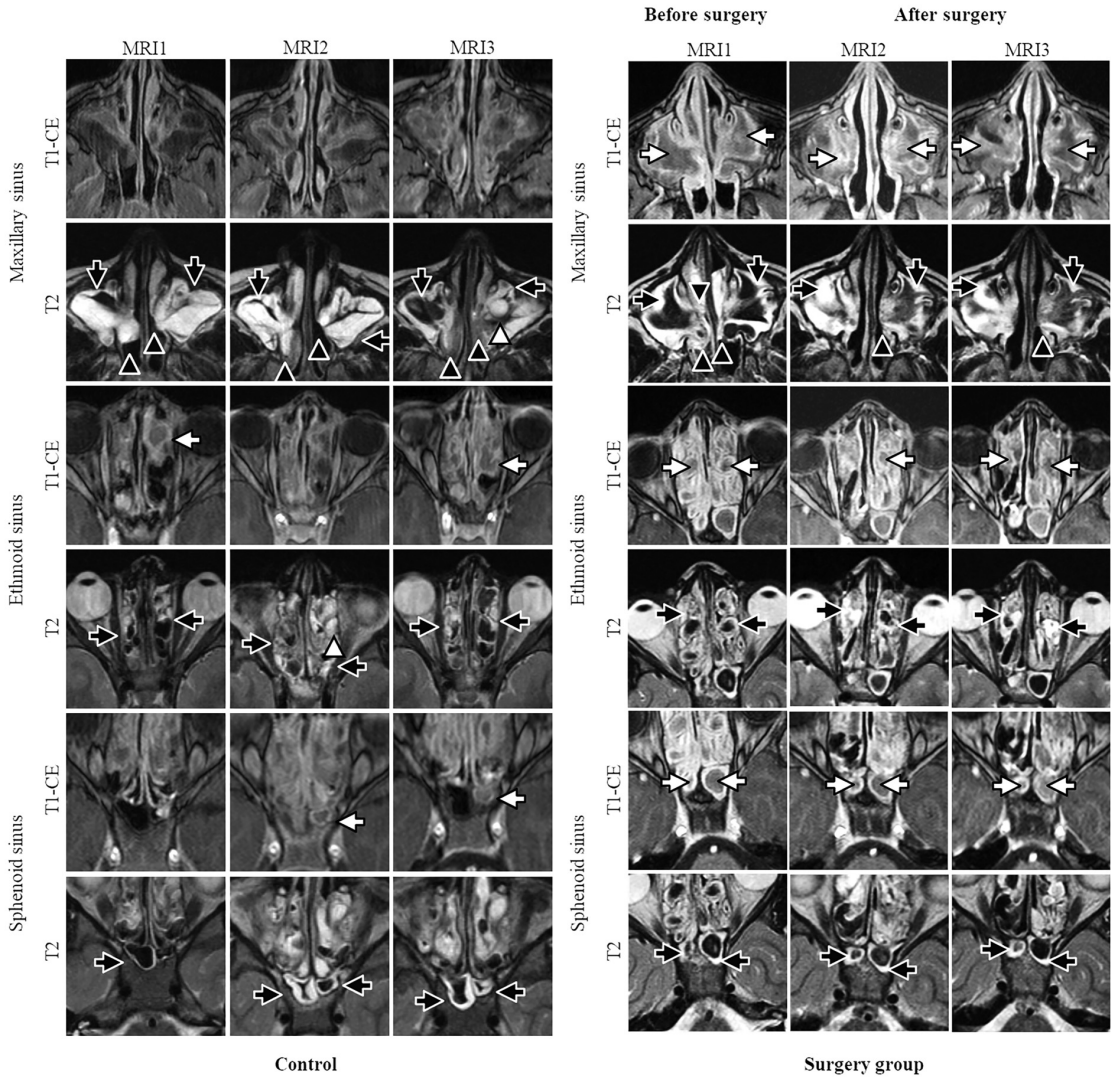
Representative annual magnetic resonance imaging (MRI) scans of an individual of the controls without sinus surgery (left panel) and of an individual of the surgery group (right panel) before (MRI1), and after surgery (MRI2, MRI3) at the ages of 9 (MRI1), 10 (MRI2), and 11 (MRI3) years. Mucosal swelling (black arrows) presents with a thickened soft tissue layer covering the inner wall of the sinus with hyperintensity on T2-weighted images. Mucopyoceles (white arrows) have a T1-weighted hyperintense filling of the sinus that do not further enhance after contrast. Typically, the center has a low or even absent signal on T2-weighted images (signal void). The soft tissue prolapse through the semilunar hiatus (maxillary sinus deformation) can be found in the maxillary sinus (black arrowhead). Polyps present with hyperintensity in T2-weighted images in the maxillary sinus and ethmoid sinus (white arrowhead). The maxillary sinus deformation in the controls remains unchanged from MRI1 to MRI3, while in the surgery group, the maxillary sinus deformation improves at MRI2 and MRI3 compared to MRI1.

The paranasal sinuses were imaged as part of the annual visit using a 1.5-T MRI scanner (Magnetom Avanto, Siemens Healthcare, Erlangen, Germany).^10^ For contrast application, T1-weighted sequences were analyzed before and after intravenous contrast application, as well as T2-weighted sequences.^9–13^ The established CRS-MRI scores^10,12^ were applied by the radiologist M.O.W., who also evaluated previous studies with excellent inter-reader reliability.^9–11^^,23^ The sinuses’ length and width were measured. The degree of opacification was assessed on a three-point scale (0: none, 1: less than 50%, 2: 50%-99%, 3: complete). Abnormalities, such as mucosal swelling, mucopyoceles, polyps, and effusion, were rated on a three-point scale (0: absent, 1: present, 2: present and dominant) (Figure 2). A score of 2 could only be assigned once per sinus and side, whereas a score of 1 could be assigned multiple times. For the maxillary sinus the presence of a semilunar hiatus deformation was assessed (0: absence of deformation; 1: prolapse without contact to the nasal septum; 2: prolapse with contact to the nasal septum) (Figure 2). The subscores were summed up to the CRS-MRI sum score, with a maximum of 68 points. Due to the low prevalence of the frontal sinuses (controls: *n* = 11, 28%; surgery group: *n* = 8, 40%), the frontal sinus subscore has been excluded from the CRS-MRI sum score for comparability.^13^ The maxillary, sphenoid and ethmoid sinuses were present in all individuals of both groups.

### Statistical Analyses

The data were analyzed using GraphPad Prism, Version 10.4.0, GraphPad Software, Boston, Massachusetts, USA. Data are presented as median, minimum-maximum range, and IQR. For the longitudinal intra-group comparisons, a repeated measures ANOVA with Bonferroni correction for related data was conducted, and for inter-group testing, a Mann–Whitney *U* test was conducted. The Fisher's exact test was utilized for the intra- and inter-group comparisons of the categorial variables prevalence and dominance. A *P*-value of <.05 was considered statistically significant.

## Results

### Soft-Tissue Prolapse Related Maxillary Sinus Width Decreases After Surgery

**Figure 3.**
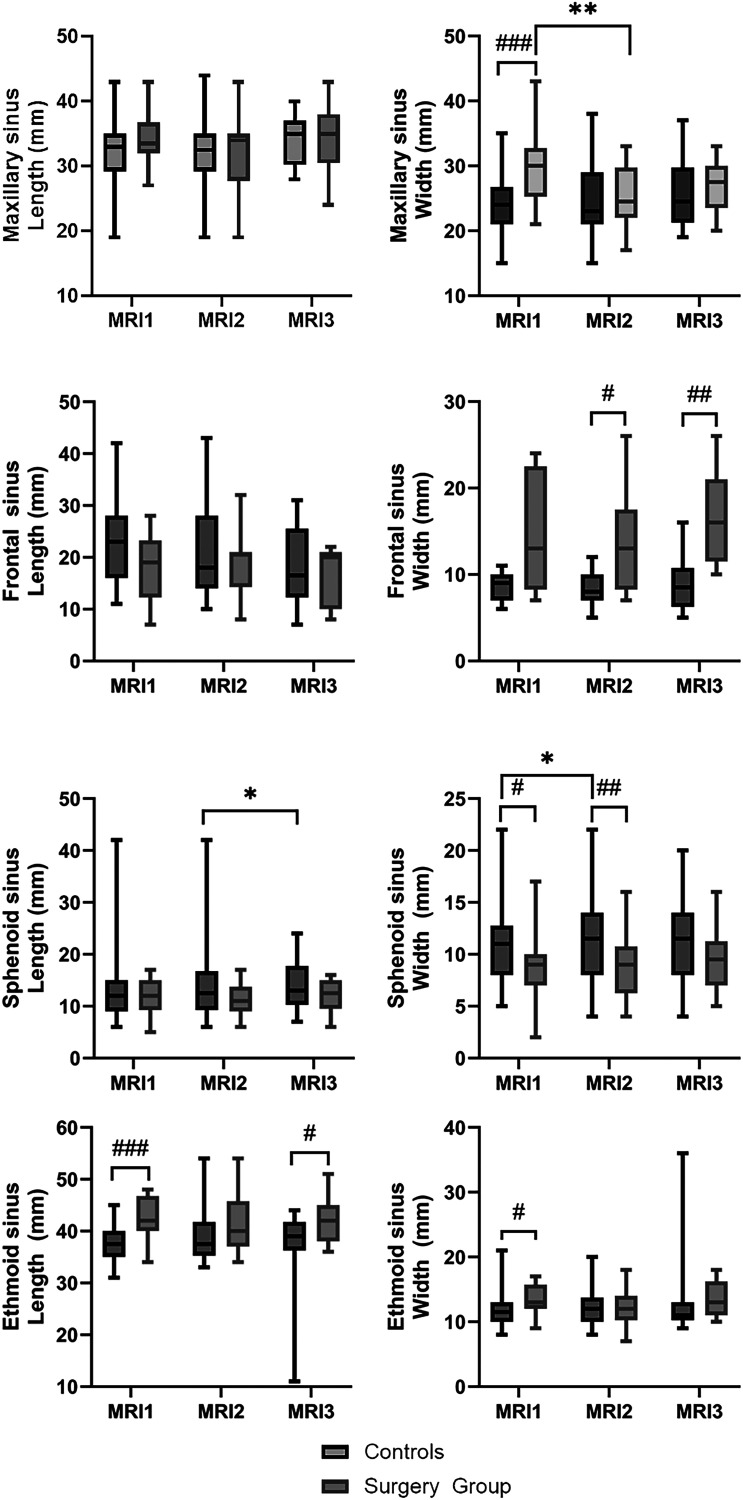
Paranasal sinus dimensions from MRI1 to MRI3 in controls and before (MRI1) and twice after (MRI2, MRI3) sinus surgery in the surgery group. Boxes present 25th to 75th percentile, the median is indicated by a horizontal line, and the whiskers mark the minimum and maximum values.

Abbreviation: MRI, magnetic resonance imaging. *P < .05. **P < .01 in the intra-group comparison. ^#^P < .05. ^##^P< .01. ^###^P < .001 in the inter-group comparison.

In controls, the maxillary sinus width was 24 mm (range: 15-35; IQR: 6) at MRI1 and remained stable from MRI1 to MRI3 (*P* = .991 to *P* > .999). In the surgery group, the maxillary sinus width was 30 mm (range: 21-43; IQR: 8) at MRI1, was significantly reduced (*P* < .01) at MRI2 (24.5 mm; range: 17-33; IQR: 8) (Figure 3) and remained stable (*P* = .544) from MRI2 to MRI3 (27.5 mm; range: 20-33; IQR: 6). At MRI1, the maxillary sinus width of the controls was significant lower compared to the surgery group (*P* < .001), while there was no difference in the maxillary sinus width between the two groups at MRI2 and MRI3 (*P* = .867 and *P* = .396, respectively) (Figure 3). The dimensions of frontal, sphenoid and ethmoid sinuses remained stable from MRI1 to MRI3 in controls (*P* = .125 to *P* > .999) and surgery group (*P* = .067 to *P* > .999) (Figure 3), except sphenoid sinus length from MRI2 to MRI3 (*P* = .040) and sphenoid sinus width from MRI1 to MRI2 (*P* = .013) in controls.

### Prevalence and Severity of Maxillary Sinus Deformation Decrease After Surgery

In controls, the maxillary (95%), frontal (64%), sphenoid (75%), and ethmoid (93%) sinuses were opacified at MRI1 (Table 2). Neither prevalences nor dominances changed from MRI1 to MRI3 for all sinuses and all abnormalities in controls (*P* = .066 to *P* > .999), except the prevalence of polyps in the maxillary sinus from MRI2 to MRI3 (+33%; *P* < .05). In the surgery group, 100% of all sinuses were opacified at MRI1 (Table 2). The prevalences and dominances for all sinuses and all abnormalities were stable from MRI1 to MRI3 (*P* = .106 to *P* > .999) in the surgery group (Table 2).

**Table 2.** Chronic Rhinosinusitis Magnetic Resonance Imaging Score Before (MRI1) and After Sinus Surgery (MRI2, MRI3) in Surgery Group Compared to Controls Without Surgery.

			**MRI1**		**MRI2**		**MRI3**	
			**Controls**	**Surgery group**	**Controls**	**Surgery group**	**Controls**	**Surgery group**
**Maxillary Sinus**	*n*		40	20	40	20	24	14
	Opacification	Prevalence, *n* (%)	38 (95)	20 (100)	34 (85)	20 (100)	22 (92)	14 (100)
	Mucosal Swelling	Prevalence, *n* (%)	38 (95)	20 (100)	35 (88)	20 (100)	22 (92)	14 (100)
		Dominance, *n* (%)	16 (40)	0 (0)^++^	11 (28)	2 (10)	3 (13)	1 (7)
	Mucopyoceles	Prevalence, *n* (%)	30 (75)	20 (100)^+^	32 (80)	19 (95)	21 (88)	13 (93)
		Dominance, *n* (%)	18 (45)	16 (80)^+^	18 (45)	14 (70)	10 (42)	10 (71)
	Polyps	Prevalence, *n* (%)	11 (28)	12 (60)^+^	15 (38)	11 (55)	17 (71)°	10 (71)
		Dominance, *n* (%)	3 (8)	4 (20)	6 (15)	4 (20)	9 (38)	2 (14)
	Effusion	Prevalence, *n* (%)	1 (3)	0 (0)	0 (0)	1 (5)	0 (0)	0 (0)
		Dominance, *n* (%)	0 (0)	0 (0)	0 (0)	0 (0)	0 (0)	0 (0)
	Deformation of semilunar hiatus	Prevalence, *n* (%)	27 (68)	19 (95)^+^	29 (73)	12 (60)*	17 (71)	11 (79)
	Subscore		12.0 (4.0-18.0; 7)	16.0 (12.0-18.0; 4)^++^	12.0 (0.0-18.0; 6)	14.0 (10.0-18.0; 4)	14.0 (7.0-18.0; 5)	16.0 (8.0-18.0; 4)
**Frontal Sinus**	*n*		11	8	15	8	12	5
	Opacification	Prevalence, *n* (%)	7 (64)	8 (100)	8 (53)	8 (100)	9 (75)	5 (100)
	Mucosal Swelling	Prevalence, *n* (%)	7 (64)	8 (100)	8 (53)	8 (100)	9 (75)	5 (100)
		Dominance, *n* (%)	1 (9)	1 (13)	2 (13)	0 (0)	1 (8)	0 (0)
	Mucopyoceles	Prevalence, *n* (%)	6 (55)	8 (100)^+^	8 (53)	8 (100)	8 (67)	5 (100)
		Dominance, *n* (%)	6 (55)	6 (75)	6 (40)	8 (100)^++^	7 (58)	4 (80)
	Polyps	Prevalence, *n* (%)	0 (0)	1 (13)	0 (0)	1 (13)	1 (8)	3 (60)
		Dominance, *n* (%)	0 (0)	0 (0)	0 (0)	0 (0)	1 (8)	1 (20)
	Effusion	Prevalence, *n* (%)	0 (0)	1 (13)	0 (0)	1 (13)	0 (0)	0 (0)
		Dominance, *n* (%)	0 (0)	1 (13)	0 (0)	0 (0)	0 (0)	0 (0)
	Subscore		0.0 (0.0-12.0; 5)	6.0 (0.0-13.0; 6)^+^	0.0 (0.0-12.0; 5)	6.0 (0.0-13.0; 6)^+^	3.5 (0.0-13.0; 6)	6.0 (0.0-13.0; 7)
**Sphenoid Sinus**	*n*		40	20	40	20	24	14
	Opacification	Prevalence, *n* (%)	30 (75)	20 (100)^+^	23 (58)	20 (100)^++^	16 (67)	14 (100)^+^
	Mucosal Swelling	Prevalence, *n* (%)	29 (73)	19 (95)^+^	23 (58)	20 (100)^++^	16 (67)	14 (100)^+^
		Dominance, *n* (%)	13 (33)	10 (50)	7 (18)	10 (50)^+^	4 (17)	4 (29)
	Mucopyoceles	Prevalence, *n* (%)	22 (55)	13 (65)	18 (45)	13 (65)	15 (63)	11 (79)
		Dominance, *n* (%)	16 (40)	10 (50)	15 (38)	10 (50)	12 (50)	10 (71)
	Polyps	Prevalence, *n* (%)	1 (3)	1 (5)	1 (3)	1 (5)	1 (4)	0 (0)
		Dominance, *n* (%)	1 (3)	0 (0)	1 (3)	0 (0)	0 (0)	0 (0)
	Effusion	Prevalence, *n* (%)	0 (0)	0 (0)	0 (0)	0 (0)	0 (0)	0 (0)
		Dominance, *n* (%)	0 (0)	0 (0)	0 (0)	0 (0)	0 (0)	0 (0)
	Subscore		8.0 (0.0-12.0; 7)	11.5 (6.0-13.0; 3)^+^	5.5 (0.0-13.0; 11)	10.5 (6.0-13.0; 3)^+^	9.0 (0.0-12.0; 9)	12.0 (6.0-12.0; 2)^+^
**Ethmoid Sinus**	*n*		40	20	40	20	24	14
	Opacification	Prevalence, *n* (%)	37 (93)	20 (100)	36 (90)	20 (100)	24 (100)	14 (100)
	Mucosal Swelling	Prevalence, *n* (%)	37 (93)	20 (100)	35 (88)	20 (100)	24 (100)	14 (100)
		Dominance, *n* (%)	35 (88)	11 (55)^++^	31 (78)	7 (35)^++^	19 (79)	3 (21)^++^
	Mucopyoceles	Prevalence, *n* (%)	23 (58)	19 (95)^++^	21 (53)	19 (95)^++^	18 (75)	14 (100)
		Dominance, *n* (%)	2 (5)	9 (45)^++^	4 (10)	9 (45)^++^	4 (17)	8 (57)^+^
	Polyps	Prevalence, *n* (%)	2 (5)	8 (40)^++^	3 (8)	10 (50)^++^	5 (21)	9 (64)^+^
		Dominance, *n* (%)	0 (0)	0 (0)	1 (3)	4 (20)^+^	1 (4)	3 (21)
	Effusion	Prevalence, *n* (%)	0 (0)	0 (0)	0 (0)	0 (0)	0 (0)	0 (0)
		Dominance, *n* (%)	0 (0)	0 (0)	0 (0)	0 (0)	0 (0)	0 (0)
	Subscore		8.0 (0.0-12.0; 4)	11.0 (8.0-14.0; 3)^++^	7.0 (0.0-12.0; 3)	11.0 (7.0-13.0; 2)^++^	9.0 (6.0-12.0; 3)	11.0 (10.0-14.0; 1)^++^
**Sum score**			28.0 (7.0-39.0; 14)	36.0 (31.0-44.0; 7)^++^	23.0 (4.0-43.0; 15)	35.0 (29.0-41.0; 8)^++^	34.0 (14.0-37.0; 13)	39.0 (30.0-42.0; 12)^+^

Prevalence and dominance *n* (%) of sinus abnormalities in the controls and surgery group are presented on a per-sinus basis, and sinus subscores and sum score are demonstrated as median (minimum-maximum; interquartile range). MRI = magnetic resonance imaging. Intra-group comparisons: * *P* < .05 versus MRI1; and ° *P* < .05 versus MRI2. Inter-group comparisons: ^+^ *P* < .05 versus controls at same MRI timepoint; and ^++^ *P* < .01 versus controls at same MRI timepoint.

In controls, the deformation of the semilunar hiatus was prevalent in 68% to 73% and was stable from MRI1 to MRI3 (*P* > .999). In the surgery group, 95% of maxillary sinuses were deformed at MRI1. This prevalence was significantly reduced at MRI2 (60%, *P* < .05) and afterwards remained stable in MRI3 (79%, *P* = .295). The surgery group had a significantly higher prevalence of semilunar hiatus deformation at MRI1 compared to controls (*P* < .05), which was no longer present after surgery at MRI2 and MRI3 (*P* = .384 and *P* = .715, respectively).

### Surgery Improves Maxillary Sinus Subscore and Deformation Score

**Figure 4.**
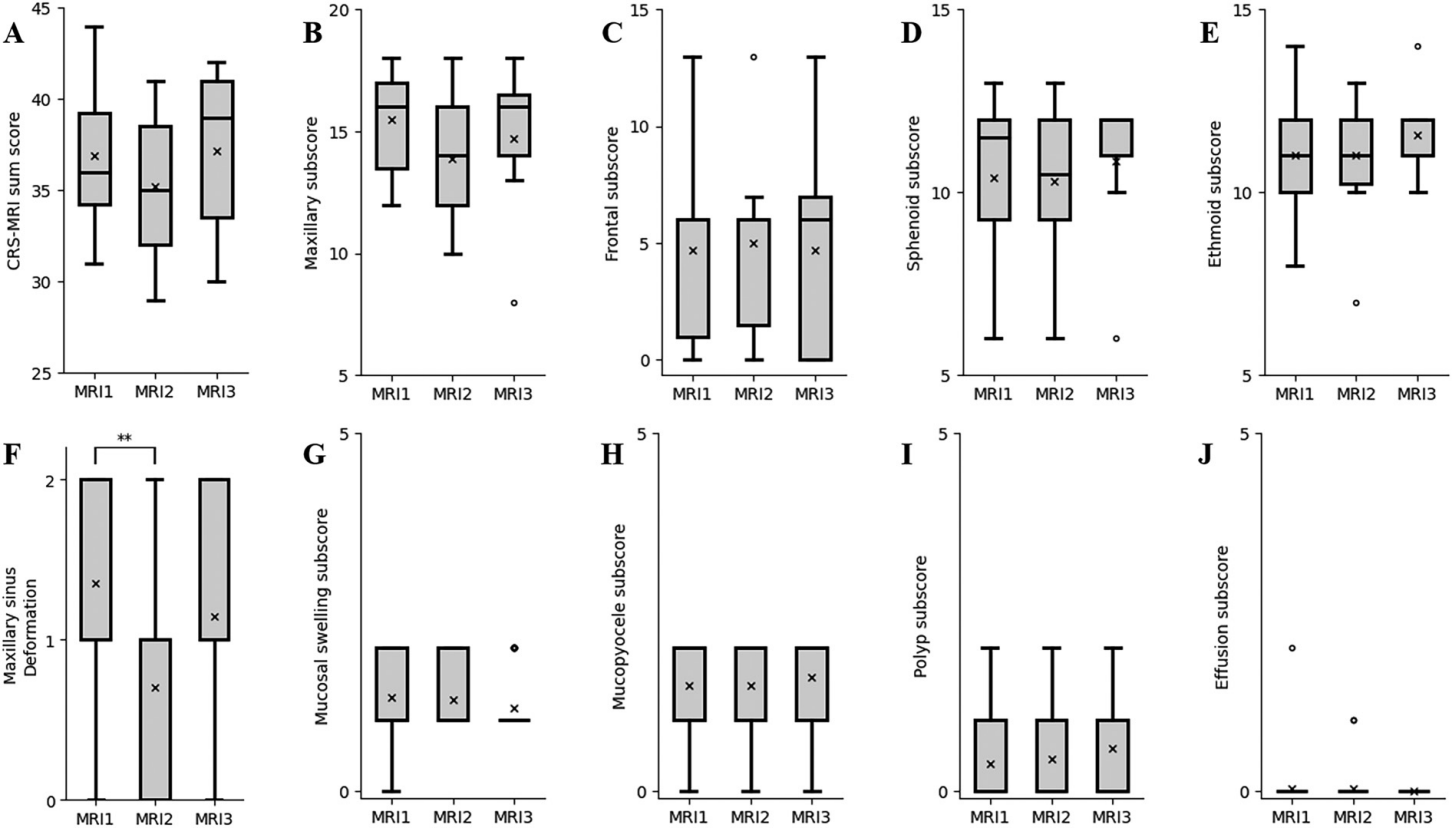
Chronic rhinosinusitis (CRS) magnetic resonance imaging (MRI) score before (MRI1) and twice after (MRI2, MRI3) surgery in patients with cystic fibrosis. The CRS-MRI sum score (A), sinus subscores (B-E) and abnormality subscores (F-J) of the surgery group were grouped by MRI timepoints. Boxes present 25th to 75th percentile, the median is presented by the horizontal line, the mean by the x and whiskers mark the minimum and maximum. Dots mark outliers.

Abbreviation: MRI, magnetic resonance imaging. *P <.05. **P < .01 in the intra-group comparison.

The maxillary sinus subscore was stable from MRI1 to MRI3 in controls (*P* = .774 to *P* > .999) and in the surgery group (*P* = .156 to *P* > .999). At MRI1, the maxillary sinus subscores were significantly higher in the surgery group (median: 16; range: 12-18; IQR: 4) compared to controls (median: 12; range 4-18; IQR: 7) (*P* < .01). After surgery, the differences in maxillary sinus subscore were no longer present between the groups (*P* = .176 to *P* = .369). The subscores of frontal, sphenoid and ethmoid sinuses were stable from MRI1 to MRI3 in the controls (*P* = .052 to *P* > .999) and in the surgery group (*P* = .517 to *P* > .999) (Figure 4, Table 2). The frontal, sphenoid and ethmoid sinus subscores were significant higher in the surgery group compared to controls for all timepoints (*P* < .001 to *P* < .05), except for frontal sinus subscore at MRI3 (*P* = .666) (Table 2).

In controls, the maxillary sinus deformation score of 0.9 (range: 0-2; IQR: 1) was stable from MRI1 to MRI3 (*P* > .999). In the surgery group, the maxillary sinus deformation subscore was 1.4 (range: 0-2; IQR: 1) at MRI1, significantly decreased at MRI2 (−0.7; *P* < .05) and remained stable between MRI2 and MRI3 (+0.4; *P* = .653) (Figure 2, Figure 4). At MRI1, the maxillary sinus deformation subscore was higher in the surgery group compared to controls (*P* < .05). After surgery, the difference in the maxillary sinus deformation subscore was no longer present between the groups (*P* = .256 to *P* = .469).

In controls, the abnormality subscores were stable from MRI1 to MRI3 (*P* = .052 to *P* > .999), except mucosal swelling subscore from MRI1 to MRI2 (−1; *P* < .05). In the surgery group, all abnormality subscores were stable from MRI1 to MRI3 (*P* = .439 to *P* > .999) (Figure 4). At all timepoints, the overall opacification subscore was significantly higher in the surgery group compared to controls (*P* < .001). The surgery group had significant higher mucopyoceles and polyps subscores at MRI1 than the controls (*P* < .001). This persisted at MRI2 (*P* < .001 to *P* < .05) and for mucopyoceles also at MRI3 (*P* < .001).

### The CRS-MRI Sum Score Was Higher in the Surgery Group and Remains Stable Despite Surgery

In controls, the CRS-MRI sum score was 28 (range: 7-39; IQR: 14) at MRI1 and remained stable at MRI2 (23; range: 4-43; IQR: 15), and MRI3 (34; range: 14-37; IQR: 13) (*P* = .860 to *P* > .999) (Table 2). In the surgery group, the CRS-MRI sum score was 36 (range: 31-44; IQR: 7) at MRI1 and remained stable at MRI2 (35; range: 29-41; IQR: 8), and MRI3 (39; range: 30-42; IQR: 12) (*P* = .743 to *P* > .999) (Figure 4). At all timepoints, the CRS-MRI sum score was significantly higher in the surgery group than in the controls (*P* < .01 to *P* < .05).

## Discussion

While previous studies have investigated the radiological impact of CFTRm on the paranasal sinuses,^11–13^^,24^ this is the first study to present a systematic assessment of the mid- to long-term impacts of sinus surgery with MRI on CRS in patients with CF. While sinus surgery may not improve overall sinus health, it can significantly reduce maxillary sinus deformation in the mid-term interval, and to a lesser extent also in the long-term interval.

Our results presented a higher disease burden in the surgery group compared to the controls and to patient cohorts from our previous studies.^11–13^ The individuals in the surgery group had a high burden of sinonasal disease as they sought surgical intervention for their condition despite having undergone optimized conservative treatment. Higher CRS-MRI scores may indicate beneficial effects of sinus surgery on CF-CRS. This has to be examined in future.

The advent of CFTRm over the past years has substantially altered the landscape of treatment options for individuals with CF. CFTRm not only improve the pulmonary manifestations of CF but also enhance sinus health in patients with CF-CRS.^2,3,6,11,13,24^ Recently, the triple combination treatment with Elexacaftor/Tezacaftor/Ivacaftor has been shown to decrease maxillary sinus deformation.^13^ Compared to studies on sinonasal outcome under CFTRm, the effect of sinus surgery on the CRS-MRI score is smaller than the previously seen effects of either Lumacaftor/Ivacaftor or Elexacaftor/Tezacaftor/Ivacaftor on the CRS-MRI score.^11–13^ Moreover, in addition to risks associated with surgery, also the acquisition of pseudomonas aeruginosa in the upper respiratory tract is a potential risk for patients with CF-CRS in the context of otorhinolaryngological surgery. The degree of opacification and the presence of specific abnormalities, such as mucosal swelling or mucopyoceles, were not significantly affected by sinus surgery in this study. As mucopyoceles can act as a reservoir for bacteria, the risk of recurrent sinus infections and complications caused by mucopyoceles remains after sinus surgery.^25–27^ Therefore, patients need to be offered a critical discussion about the potential disadvantages and benefits of sinus surgery based on their individual symptom burden, and informed about the expected long-term course after surgery and the continued increased risk of relapse of CF-CRS.^17,28^

Various surgical approaches have been described for sinus surgery in patients with CF-CRS.^17^ The aim is to create wide sinonasal openings and drainage pathways.^22^ This improves nasal irrigation, topical treatment and drainage of sinonasal fluids, and prevents the formation of bacterial reservoirs.^15,29–31^ Some surgeons perform minimally invasive functional endoscopic sinus surgery limited to the main affected structures.^27,32^ Other surgeons perform extended procedures, as they are said to improve long-term surgical outcomes.^27^ Previously, intranasal ethmoidectomy, Caldwell-Luc procedures and medial maxillectomy with and without inferior turbinate reduction, as well as frontal sinus trephination, extensive mucosal and polyp resections, and Draf procedures have been described as techniques for sinus surgery in patients with CF-CRS.^15,27,31,33–39^ In the authors’ opinion, radical resections do not offer any additional benefits, but cause more scar tissue and carry an elevated risk of surgical complications such as injury to the orbital structures or skull base. For this reason, the authors performed a semi-radical approach,^18,40–42^ in which wide sinus openings are created without extensive techniques such as medial maxillectomies or Draf procedures.

While the CRS-MRI score did not detect benefits of sinus surgery on the frontal, sphenoid, and ethmoid sinuses and on nearly all abnormality and sinus subscores, sinus surgery has a notable effect on maxillary sinus width and deformation. The surgery group presented with a significant higher preoperative maxillary sinus width and prevalence of maxillary sinus deformation than the controls. This leads to airway obstruction and, consequently, to symptomatic CF-CRS,^22^ which again may explain why patients in the surgery group were more likely to undergo sinus surgery. A significant maxillary sinus deformation detected in MRI might serve as a future marker for a potential beneficial effect of sinus surgery in CF-CRS, even in patients receiving CFTRm, as they may still experience relevant nasal airway obstruction. However, the actual correlation between these structural abnormalities and clinical symptoms remains unclear up to now and has yet to be investigated.

The present study has some limitations. First, the number of patients included was limited. It is possible that most individuals with CF have adapted to their sinonasal condition from infancy without subjective symptoms or non-surgical treatment options have been optimized,^9,10,43,44^ which may explain the low number of individuals with CF undergoing sinus surgery. Second, as the focus of this study was on the radiological assessment of the effects of sinus surgery on CF-CRS, postoperative clinical outcomes such as the need for sinonasal-related symptomatic treatment or antibiotics or clinical scores such as the sinonasal outcome test-22 (SNOT-22) were not assessed.

In conclusion, our study represents the first systematic investigation of the radiological outcome of sinus surgery in individuals with CF-CRS based on the CRS-MRI score. MRI is a suitable tool for assessing structural changes of CF-CRS following sinus surgery. Our results suggest an improvement of maxillary sinus deformation by sinus surgery, at least temporarily in the mid-term and to a lesser extent also in the long-term interval of 2 years. The decision to proceed with surgery should be based on clinical symptoms, and can be supported by MRI.

## Supplemental Material

sj-docx-1-ajr-10.1177_19458924261435339 - Supplemental material for Sinus Surgery Improves Maxillary Sinus Deformation in Cystic Fibrosis Detected by Magnetic Resonance Imaging Supplemental material, sj-docx-1-ajr-10.1177_19458924261435339 for Sinus Surgery Improves Maxillary Sinus Deformation in Cystic Fibrosis Detected by Magnetic Resonance Imaging by Miray-Su Yılmaz Topçuoğlu, Ingo Baumann, Veronika Kolb, Hans-Ulrich Kauczor, Olaf Sommerburg, Mirjam Stahl, Marcus A Mall, Monika Eichinger, Lena Wucherpfennig and Mark O Wielpütz in American Journal of Rhinology & Allergy
