# Supplementary material for: Sinus Surgery Improves Maxillary Sinus Deformation in Cystic Fibrosis Detected by Magnetic Resonance Imaging
Source: Am J Rhinol Allergy. 2026 Apr 6;40(5):362–73. doi: 10.1177/19458924261435339 (PMC13451674; doi:10.1177/19458924261435339)
Supplement: Supplementary file 1 [file sj-docx-1-ajr-10.1177_19458924261435339.docx]

# Supplement

**Supplemental Table E1. Summary of *CFTR* genotypes of controls and surgery group, grouped according to pancreatic status.**

| **Controls** | | | | **Surgery group** | | |  |
| --- | --- | --- | --- | --- | --- | --- | --- |
| **Pancreatic insufficiency** | | **Pancreatic sufficiency** | | **Pancreatic insufficiency** | | **Pancreatic sufficiency** | |
| ***CFTR* Genotype** | **Patients, n** | ***CFTR* Genotype** | **Patients, n** | ***CFTR* Genotype** | **Patients, n** | ***CFTR* Genotype** | **Patients, n** |
| Total | 18 | Total | 2 | Total | 8 | Total | 2 |
| F508del /F508del | 7 | F508del /F508del | 1 | F508del /F508del | 2 | F508del/R347P | 1 |
| F508del/N1303K | 1 | F508/D1152H | 1 | F508del/R553X | 2 | F508del/p.T1299I | 1 |
| F508del/CFTRdele25-27 | 1 |  |  | F508del/Q220X | 1 |  |  |
| F508del/Q552X | 1 |  |  | F508del/G542X | 1 |  |  |
| F508del/Q39X | 1 |  |  | 306insA/R347P | 1 |  |  |
| F508del/1870delG | 1 |  |  | unknown | 1 |  |  |
| F508del/2183AA>G | 1 |  |  |  |  |  |  |
| F508del/G551D | 1 |  |  |  |  |  |  |
| F508del/M1101K | 1 |  |  |  |  |  |  |
| F508del/3821delT | 1 |  |  |  |  |  |  |
| F508del/R851X | 1 |  |  |  |  |  |  |
| R553X/R553X | 1 |  |  |  |  |  |  |

**Supplemental Table E1. Summary of *CFTR* genotypes of controls and surgery group, grouped according to pancreatic status.**
CFTR = cystic fibrosis transmembrane conductance regulator.
